# The role of polyphenols in overcoming cancer drug resistance: a comprehensive review

**DOI:** 10.1186/s11658-021-00301-9

**Published:** 2022-01-03

**Authors:** Parisa Maleki Dana, Fatemeh Sadoughi, Zatollah Asemi, Bahman Yousefi

**Affiliations:** 1 Research Center for Biochemistry and Nutrition in Metabolic Diseases, Institute for Basic Sciences, Kashan University of Medical Sciences, Kashan, Islamic Republic of Iran; 2 Molecular Medicine Research Center, Tabriz University of Medical Sciences, Tabriz, Iran; 3 Department of Biochemistry, Faculty of Medicine, Tabriz University of Medical Sciences, Tabriz, Iran

**Keywords:** Polyphenols, Curcumin, Resveratrol, Epigallocatechin gallate, Chemoresistance

## Abstract

Chemotherapeutic drugs are used to treat advanced stages of cancer or following surgery. However, cancers often develop resistance against drugs, leading to failure of treatment and recurrence of the disease. Polyphenols are a family of organic compounds with more than 10,000 members which have a three-membered flavan ring system in common. These natural compounds are known for their beneficial properties, such as free radical scavenging, decreasing oxidative stress, and modulating inflammation. Herein, we discuss the role of polyphenols (mainly curcumin, resveratrol, and epigallocatechin gallate [EGCG]) in different aspects of cancer drug resistance. Increasing drug uptake by tumor cells, decreasing drug metabolism by enzymes (e.g. cytochromes and glutathione-S-transferases), and reducing drug efflux are some of the mechanisms by which polyphenols increase the sensitivity of cancer cells to chemotherapeutic agents. Polyphenols also affect other targets for overcoming chemoresistance in cancer cells, including cell death (i.e. autophagy and apoptosis), EMT, ROS, DNA repair processes, cancer stem cells, and epigenetics (e.g. miRNAs).

## Introduction

Localized solid tumors are often treated with surgery in their early stages. However, other treatment modalities are applied at advanced stages and/or following the surgery, such as targeted therapies, radiotherapy, immunotherapy, and chemotherapy [1]. Advances made in the field of antitumor agents have led to a significant increase in patients' life quality and disease-free survival [2]. Despite the importance of chemotherapeutic drugs, there are significant drawbacks in using them to treat cancer, such as solubility and instability of drugs, nonspecific drug delivery, and adverse effects due to systemic toxicity [3]. Furthermore, recurrence and relapse of cancer occur in some patients even after a favorable response at the beginning of the treatment. Indeed, acquired drug resistance has become an important challenge that results in the failure of cancer treatment [2]. Both acquired and intrinsic processes can lead to chemoresistance in cancer cells [4]. Acquired drug resistance indicates a newly developed resistance against a therapeutic approach that was effective at the beginning. Intrinsic chemoresistance involves a pre-existing factor that causes a drug to be inefficient [5]. Tumor cells’ heterogeneity is one of the factors leading to chemoresistance. Stem-like cancer cells are renewable subpopulations of tumor cells that are responsible for heterogeneity. There are various cell generations within one tumor, and each clone is sensitive to chemotherapeutic agents to some degree. Therefore, targeting tumor cells with a single agent may not lead to a favorable response [6–8]. Increased drug efflux, changes in the target of drugs, apoptosis, and repair signaling pathways are other mechanisms involved in the resistance of cancer cells to chemotherapeutic drugs [4, 9].

## Polyphenols and their therapeutic application in cancer

**Fig. 1:**
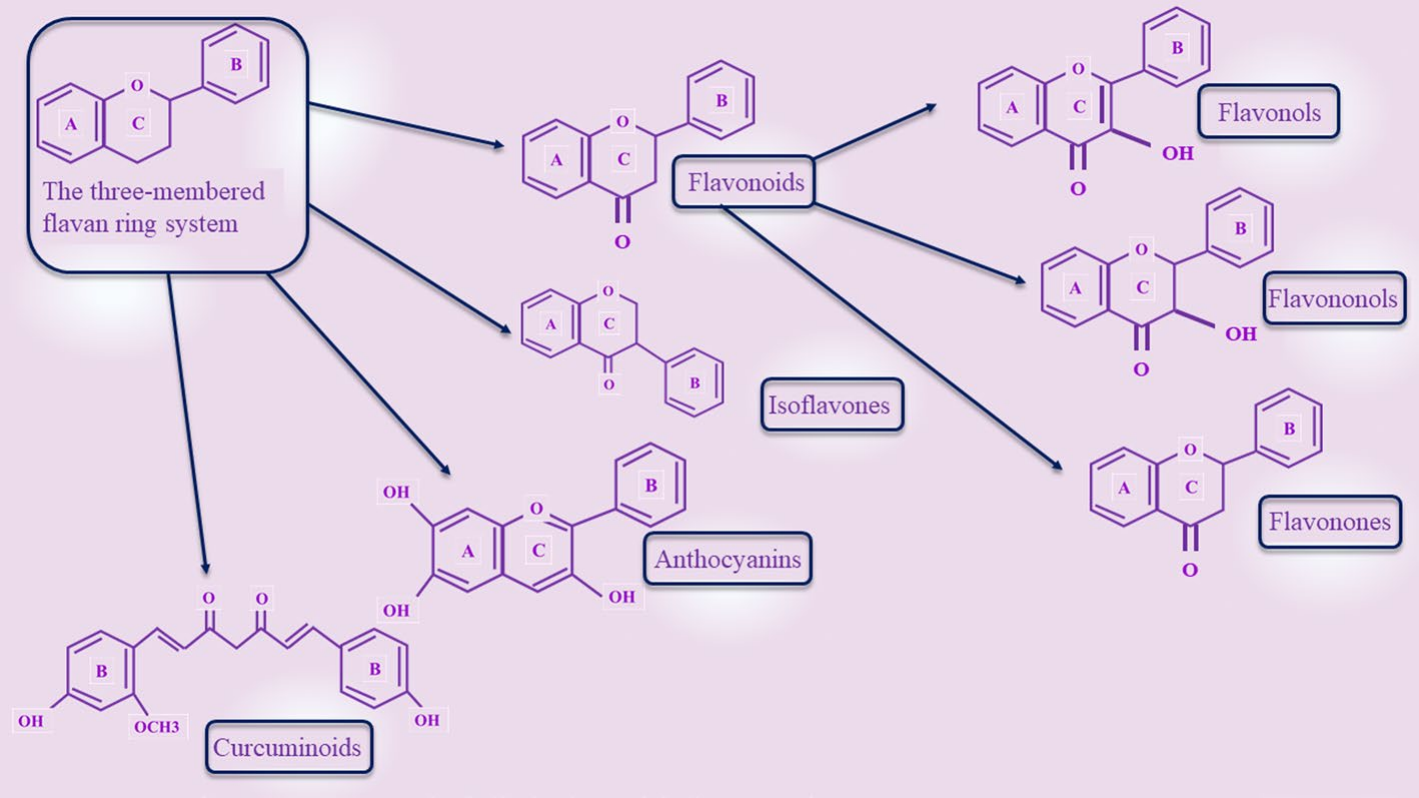
Schematic representation of different structures of polyphenols. These agents have a three-membered flavan ring system in common

Polyphenols are a large family of 10,000 plant compounds that are known for their common structural features including the three-membered flavan ring system and multiple phenol units [10, 11]. These natural compounds are mostly found in fruits, green and black tea, coffee, red wine, cocoa, and seeds [12]. These beneficial organic agents are categorized into several subclasses including catechins, flavonoids (which contain flavonols, flavanols, and flavones), anthocyanins, catechins, isoflavones, chalcones, curcuminoids, and phenolic acids (structures are shown in Fig. 1) [12, 13].

**Fig. 2:**
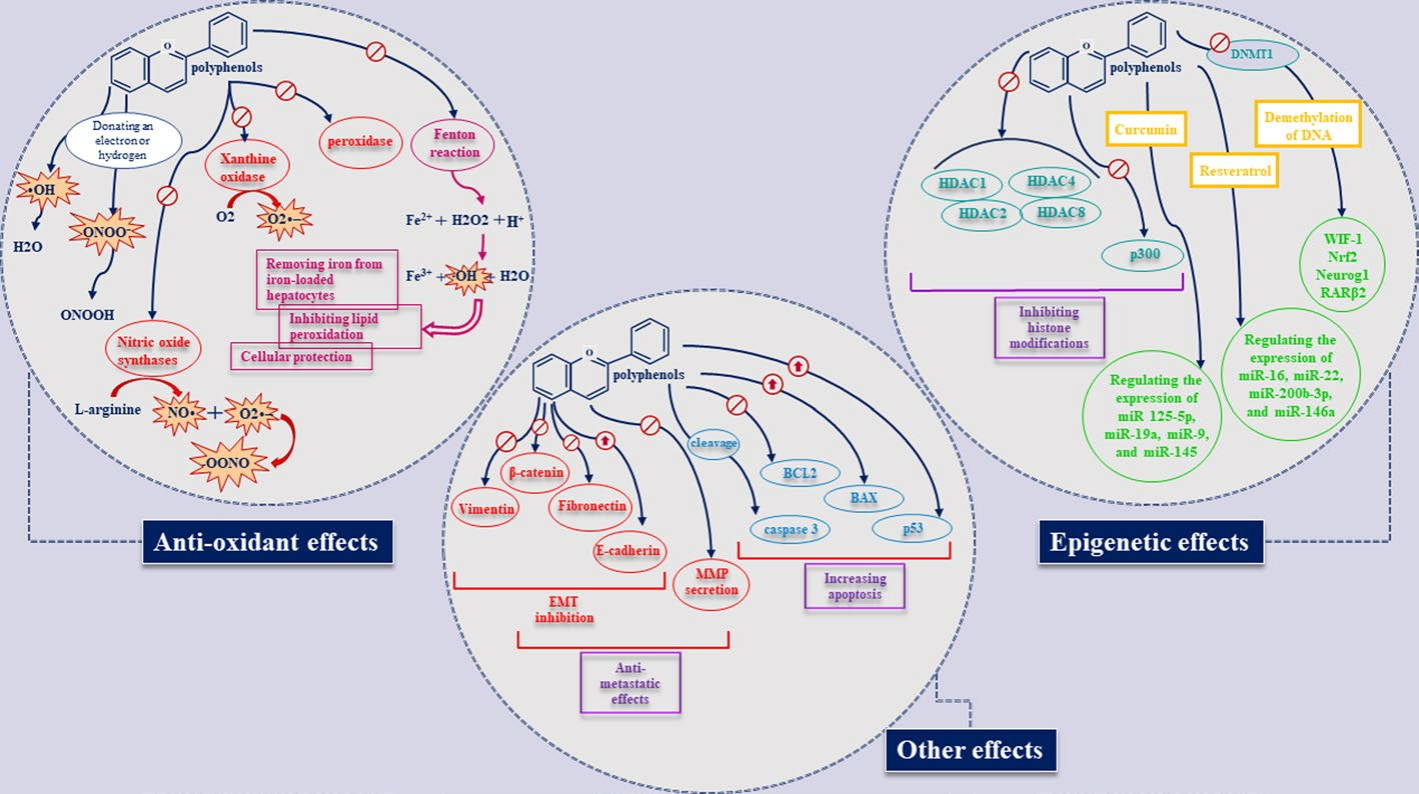
Some of the anti-cancer effects of polyphenols including epigenetic, anti-metastatic, pro-apoptotic, and anti-oxidant impacts

The idea of using polyphenols for treating cancer patients is not new. Early studies considering the anti-cancer effects of different polyphenols were conducted in the late twentieth century and our knowledge on these advantageous agents has been widely improved since then [14, 15]. What makes these agents greatly beneficial and interesting is that they attack cancer cells in a variety of ways and confront many cancer hallmarks (summarized in Fig. 2). Therefore, we shall briefly discuss different aspects of polyphenols’ effects in this section.

### Antioxidant effects

The anti-oxidant impacts of polyphenols are possible through either scavenging free radicals or building a barrier against their generation (Fig. 2) [10]. The main free radicals that exist in our cells and cause oxidative stress are reactive oxygen species (ROS) and reactive nitrogen species (RNS) [16]. The former mechanism of polyphenols’ action relies on the presence of benzene ring-bound hydroxyl groups which provide the ability to donate a hydrogen atom or an electron to free radicals [17]. This occurrence stabilizes free radicals and prevents them from damaging the cellular components [17]. It seems that the B ring of polyphenols plays the most important role in scavenging hydroxyl, peroxyl, and peroxynitrite radicals [17]; however, the scavenging property can also be dependent on other structural parts in different polyphenols. For instance, in flavonoids, which are the best known polyphenols, a free 3-OH is mostly responsible for neutralizing the free radical [18].

As mentioned above, polyphenols also have the capacity to inhibit the generation of ROS and RNS by interfering with the enzymes involved in their production. Nitric oxide synthases (NOS), xanthine oxidase (XO), and peroxidase are some of these enzymes, whose activity can be altered when certain interactions occur between them and polyphenols [19, 20]. Xanthine oxidase is one of the most important enzymes that generate superoxide from oxygen molecules [21]. Quercetin, kaempferol, myricetin, and chrysin are among the polyphenols that are confirmed to inhibit this enzyme [22]. NOS is also essential for producing nitric oxide in endothelial cells and macrophages. Nitric oxide mediates oxidative stress by increasing the production and concentration of peroxynitrite and thereby damaging the cellular membrane [20]. Anthocyanidins are a subclass of polyphenols that prevent NOS from generating nitric oxide and thereby repressing their **.**NO scavenging capacity [23].

Another production mechanism that is prone to be affected by the chelating properties of polyphenols is the metal-mediated reduction of peroxides [24]. In this mechanism, which is known as the Fenton reaction, Fe^2+^ ions reduce H_2_O_2_ and thereby create a hydroxyl radical which is harmful to cells [24, 25]. In this concentration-dependent process, polyphenols can interfere as chelating agents and form stable complexes with iron [24]. Inhibiting lipid peroxidation, removing iron from iron-loaded hepatocytes, blocking the Fenton reaction, and cellular protection are the results of the interactions between diverse polyphenols and iron [25–28].

### Effects on apoptosis

As regards apoptosis, a great number of polyphenols are able to induce cell death by altering the expression of apoptosis-related genes. Curcumin is one of the most investigated polyphenols, which increases apoptosis in cancer cells through a variety of mechanisms, for instance, diminishing the intracellular ROS, phosphorylation, and activation of mitogen-activated protein kinase (MAPK) signaling pathway [29], increasing intracellular Ca influx and activating calcium/calmodulin-dependent protein kinase II (CaMKII) signaling [30], increasing PI3K/Akt protein expression (31), inducing p53 expression (32), regulating miRNAs [33], reducing the levels of B-cell lymphoma 2 (BCL2), inducing BCL-2-associated X protein (BAX), and cleaving caspase 3 [34]. Resveratrol is another beneficial polyphenol that is observed to have an effect on apoptosis in many types of cancer including bladder [35], prostate [36], breast, lung, glioblastoma, colon, and ovarian [37–42]. For instance, resveratrol suppresses proliferation and migration of ovarian cancer SKOV3 and A2780 cells. Also it impairs glycolysis and induces apoptosis. Evidence shows that treating cells with resveratrol reduces both activation and expression of mTOR and downstream kinase of AMPK while increasing the activation and expression of caspase-3 and AMPK. In vivo findings also indicated that resveratrol inhibits the growth of ovarian cancer as well as liver metastasis in a mouse xenograft model [38]. In vitro findings demonstrate that EGCG inhibits the viability of oral squamous cell carcinoma HSC-3 cells. Moreover, it induces cell cycle arrest at the G1 phase. EGCG has also been shown to significantly increase the activity of caspase-3 and -7 as well as apoptotic cells. In vivo investigations on mice xenograft models indicated that EGCG leads to a 42.5% reduction in the size of the tumor compared with the control group. Furthermore, the percentage of apoptotic cells is higher in mice treated with EGCG [43].

### Changes in cell cycle and inhibition of proliferation

Cell cycle arrest is another anti-cancer effect of these plant compounds which is exerted by resveratrol, curcumin, and diverse flavonoids in cancer cells [40, 44, 45].

### Anti-metastatic effects

Metastasis is defined as a series of concurrent mechanisms which help the tumor cells gain the ability to migrate from their primary site to other sites of the body and increase the cancer lethality [46]. Metastasis occurs as a result of the effects of microenvironmental ingredients such as stromal fibroblasts and immune cells on the tumor cells. Cellular motility, hypoxia, EMT, and angiogenesis are the primary mechanisms that prepare tumoral cells for infiltration [47–49]. According to research, matrix metalloproteinases (MMPs), TGF-β, and TP53 have essential roles in managing metastasis [47–49].

Polyphenols have shown their capabilities in influencing several steps of this process. For instance, curcumin affects the EMT-related proteins including vimentin, fibronectin, β-catenin, and E-cadherin along with the genes expressed in cancer stem cells such as Oct4, Nanog, and Sox2, and thereby decreases the metastatic features of cancer cells [50]. Quercetin and its derivatives are also effective for inhibiting EMT, MMP secretion, NF-kappaB, and migration of the cancer cells and thus metastasis [51–55]. Reversing EMT through AKT/GSK-3β/Snail signaling and diminishing the levels of MMP-2 and 9, and Smad2 and 3 are the anti-metastasis effects of resveratrol [56, 57].

### Epigenetic effects

Epigenetic dysregulations and abnormalities are the basis of tumor initiation, progression, and resistance to therapy [58]. DNA methylation, histone modifications, chromatin/nucleosome remodeling, and miRNA regulation are some of the epigenetic alterations which are involved in a variety of cancer features [58].

Curcumin is one of the most efficient polyphenols in preventing these alterations from aiding the cancer cells. Histone deacetylases (HDACs) are a class of gene silencing-related enzymes that diminish the number of acetyl groups from histones [59]. One of curcumin’s effects is to inhibit these enzymes and thereby regulate the proliferation and apoptosis of various cancer cells [60]. HDAC1, HDAC2, HDAC3, HDAC4, and HDAC8 can be inhibited by curcumin [60–63]. Histone acetyltransferases (HATs) are another class of enzymes that also predict cancer cell growth and survival. One of these enzymes is p300, which has been shown by some investigations to be inhibited by curcumin, through either a direct or indirect manner [64, 65].

Furthermore, curcumin suppresses DNA methylation in the promoter region of many cancer-related genes, including the tumor suppressor gene Wnt inhibitory factor-1 or WIF-1 [66], FANCF [67], Nrf2 [68], Neurog1 [69], and RARβ2 [70] through decreasing the DNA methyltransferase 1 level (DNMT1) [71, 72].

Regulating the amounts of microRNAs is a newly discovered aspect of curcumin’s effect in the field of cancer therapy which we have discussed in detail in our previous paper [73]. miR-125-5p, miR-19a, miR-9, and miR-145 are some of the miRNAs affected by curcumin in nasopharyngeal, breast and ovarian cancers and leukemia [74]. Regarding other polyphenols, resveratrol is also able to modulate miR-200, miR-122-5p, miR-20, and miR-633 [75–78]. miR-16, miR-22, miR-200b-3p, and miR-146a are some of the miRNAs regulated by quercetin in cancerous cells [79–82]. Epigallocatechin-3-gallate, genistein, and DIM also contribute to reversing epigenetic alterations in cancer cells via diverse microRNAs [83, 84].

## Polyphenols modulate cancer drug resistance

Several studies have reported that polyphenols can affect different aspects of cancer drug resistance. Herein, we provide a brief discussion on how each mechanism changes the sensitivity of cancer cells to chemotherapeutic drugs. Furthermore, we review the literature on the role of polyphenols (mainly curcumin, resveratrol, and EGCG) in overcoming cancer drug resistance by each of these mechanisms.

### Drug uptake by tumor cells

Facilitation of diffusion, passive transfer, and active transport are different types of drug absorption to the tumor cells [85]. Decreased drug uptake is a mechanism by which tumor cells develop chemoresistance against therapeutic agents [86]. The reduced tendency for binding to drugs is a common process that leads to decreased drug absorption. Another mechanism is decreased number of transporters [87]. Drug formulations based on nanotechnology have attracted a lot of attention in recent years due to various reasons, such as targeted drug delivery, ability to encapsulate multiple agents, higher biocompatibility, decreased side effects, and slow release rate. Another important advantage of nano-formulations is the ability to enhance bioavailability of drugs and overcome chemoresistance [88]. Among other beneficial effects, nano-formulations lead to an increase in cellular drug uptake. Several studies have been conducted on the role of polyphenols in designing nanomaterials for drug delivery. Tsai and colleagues prepared gold nanoparticles based on gelatin-doxorubicin (DOX) and EGCG to suppress the growth of prostate cancer. They reported that Au nanoparticles that are coated with EGCG and gelatin-DOX efficiently deliver DOX through the laminin 67R receptor and increase the cellular uptake of the drug [89]. Studies have also demonstrated that using EGCG leads to the reduction of Au^3+^, providing enhanced Au nanoparticles that show higher drug uptake by cancer cells [90–92].

Reduced intracellular accumulation of platinum-based antitumor agents (e.g. cisplatin) has been associated with chemoresistance of tumors. Proteins playing a role in the hemostasis of copper are reported to be transporters of platinum. Copper transporter 1 (CTR1) is the main influx transporter of copper which is involved in the resistance to platinum [93]. Wang et al. [94] reported that EGCG increases the expression of CTR1 at mRNA and protein levels in ovarian cancer cells as well as xenograft mice. Indeed, EGCG treatment suppresses the rapid cisplatin-induced degradation of CTR1 and enhances the cellular accumulation of cisplatin and DNA-Pt adducts. This causes an increase in the sensitivity of OVCAR3 and SKOV3 ovarian cancer cells to cisplatin [94]. Another study also showed that EGCG upregulated CTR1 while increasing the accumulation of platinum in non-small cell lung cancer (NSCLC) cells (including H460, H1299, and A549), a xenograft model of nude mice, and cisplatin (cDDP)-resistant A549 cells. It was found that hsa-mir-98-5p inhibits expression of the CTR1 gene. Meanwhile, lncRNA nuclear enriched abundant transcript 1 (NEAT1) increases the expression of NEAT1. Indeed, NEAT1 is suggested to increase EGCG-induced CTR1 through sponging hsa-mir-98-5p. Therefore, EGCG enhances the sensitivity of NSCLC cells to cisplatin both in vitro and in vivo [95]. MRP1 regulates both absorption and disposition of various xenobiotic and endogenous substrates, such as drugs, across different physiological barriers [96]. In DOX-resistant acute myeloid leukemia (AML)-2/DX300 cells, the expression level of the MRP1 gene is reported to be higher compared to the wild-type AML-2/WT cells. Treating this chemoresistant cell line with resveratrol leads to a reduction in MRP1 expression. Furthermore, the absorption of the MRP1 substrate 5(6)-carboxyfluorescein diacetate is reported to be enhanced by resveratrol. Therefore, it is suggested that resveratrol may enhance DOX cellular absorption by reducing the gene expression of MRP1 [97].

### Drug efflux

The main cause of multi-drug resistance in cancer cells is the increased efflux of antitumor agents through drug transporters which are embedded in the membrane [98]. P-glycoprotein (p-gp/ABCB1/MDR1) is a member of the ATP-binding cassette (ABC) superfamily which acts as a drug transporter in humans. Multidrug resistance protein 1 (MRP1/ABCC1) and breast cancer resistant protein (BCRP/BCP/MXR/ABCG2) are other well-studied drug transporters of the ABC superfamily [99–102].

P-gp is a pump involved in drug efflux and is associated with multi-drug resistance [103]. In response to chemotherapeutic agents, p-gp is upregulated, which, in turn, leads to a reduction in the drugs’ intracellular accumulation and decreases the efficacy of drugs [104]. Didox is a polyhydroxyphenol that serves as a chemo-sensitizer. In HCT 116 colorectal cancer cells, the combination of didox and resveratrol with DOX leads to the reduction of DOX IC_50_ from 0.96 ± 0.02 μM to 0.4 ± 0.06 μM and 0.52 ± 0.05 μM, respectively. Both didox and resveratrol significantly increase DOX intracellular entrapment by preventing the efflux effect of p-gp [105]. EGCG is shown to modulate p-gp function and enhance the intracellular entrapment of DOX in drug-resistant KB-A1 cells. Indeed, combination of 50 μM of EGCG with 10 μM DOX for 4 h increases the DOX intracellular concentration by 2.3 times in KB-A1 cells compared to treatment with DOX alone*.* Furthermore, in vitro studies and xenograft models confirmed that EGCG enhances the antitumor activities of DOX in drug-resistant tumors [106]. Liang et al. also reported that EGCG or epicatechin gallate (ECG) at higher doses slightly suppressed the proliferation of resistant human hepatocellular carcinoma (HCC) BEL-7404/DOX cells in vitro and in vivo. Meanwhile, lower doses of the mentioned compounds with DOX lead to significant suppression of HCC cell proliferation in vitro as well as growth of hepatoma in a mouse xenograft model compared to administration of either agent alone. Using EGCG or ECG in combination with DOX increases the intracellular accumulation of DOX, suggesting that catechins suppress the activity of the P-gp efflux pump. Furthermore, this combinational treatment enhanced the intracellular retention of a P-gp substrate, rhodamine 123, while reducing the expression of mRNA of HIF-1α and MDR1 [107].

Research has shown that some cancer cells are dependent on the PI3K/Akt/mTOR pathway in order to survive following DNA damage. Therefore, the repair of DNA damage can be inhibited by suppressing this signaling pathway, which, in turn, increases the sensitivity to radio- and chemotherapy [108]. In K562/ADR cells, overexpression of p-gp reduces the cytotoxic effects of antitumor agents and consequently multi-drug resistance occurs. Treating K562/ADR cells with a combination of resveratrol and bestatin results in a decrease in bestatin’s IC_50_ values and enhances the bestatin-induced apoptosis. Resveratrol exerts this potentiating effect by suppressing the activity of p-gp and lowering mRNA and protein levels of p-gp. In addition, resveratrol reduces the phosphorylation of mTOR and Akt without affecting ERK1/2 and JNK. Thus, it is implied that the resveratrol inhibitory effect on p-gp is mediated by inhibition of the PI3k/Akt/mTOR pathway [109].

Through active drug efflux, MRP5 causes drug resistance against gemcitabine and 5-fluorouracil (5-FU). Curcumin is shown to increase the sensitivity of MRP5 over-expressing HEK293, PANC-1, and MiaPaCa-2 cells to 5-FU. Therefore, it is suggested that curcumin can act as an inhibitor of MRP5 while reversing multidrug resistance in pancreatic cancer [110]. Curcumin increases DOX anticancer effects in DOX-resistant breast cancer MDA-MB-231/DOX and MCF-7/DOX cell lines [111]. It is observed that curcumin treatment leads to an increase in DOX intracellular accumulation. The mentioned effect is negatively associated with the activity of ATP binding cassette subfamily B member 4 (ABCB4). Treating cells overexpressing ABCB4 with DOX and curcumin reduces DOX efflux. In addition, ABCB4 ATPase activity is suppressed by curcumin without any changes in its protein expression (111).

### Alteration in drug metabolism

Evidence has indicated that following treatment with antitumor agents, some cell-protective gene products are induced. In phase I and phase II of drug metabolism, some enzymes are involved which are useful for the detoxification from harmful endogenous and exogenous compounds. In phase I of drug metabolism, different cytochrome isoforms are involved, including CYP1A2, CYP1A6, CYP1B1, CYP2B6, and CYP2C19 [112]. It is reported that curcumin suppresses the activity of CYP3A while increasing the rhodamine-123 intracellular accumulation in MCF-7/ADR cells that overexpress p-gp. Curcumin treatment also significantly increases the bioactivity of tamoxifen. It is suggested that curcumin may suppress the cytochrome-mediated metabolism of tamoxifen to 4-hydroxyfamoxifen, an active metabolite of tamoxifen, as evidenced by the reduced metabolite-parent AUC ratio. Therefore, curcumin-mediated enhanced bioavailability of tamoxifen is probably mediated by the suppression of tamoxifen metabolism in the liver and the small intestine [113].

Gamma-glutamyl transferases (GGTs), thiopurine methyltransferases (TPMTs), glutathione-S-transferases (GSTs), dihydropyrimidine dehydrogenases (DPDs), and uridine diphospho-glucuronosyltransferases (UGTs) are enzymes playing roles in phase II of drug metabolism. The altered expression of these enzymes may cause multidrug resistance in cancer cells [114, 115]. Curcumin is shown to reduce the activity of gamma-glutamyl transpeptidase (GGTP) in ZR-75-1 mammary cells which are resistant to oxidative damage [116]. Studies have reported that a moderate reduction in the levels of glutathione can enhance the sensitivity of tumor cells to chemotherapeutic agents [117]. GSTs are observed to be overexpressed in different cancers (e.g. cancers of breast, liver, and lung) and lead to drug resistance [118–120]. Therefore, suppressing GST is suggested to help overcome cancer resistance to chemotherapeutic drugs. Derivatives of flavonoids (e.g. baicalin, phloretin, baicalein, and phloridzin) are reported to be related to the suppression of GST functions [121]. Curcumin and ellagic acid are capable of inhibiting GSTs M1-1, M2-2, A1-1, A2-2, and P1-1 while using 1-chloro-2,4 dinitrobenzene (CDNB) as a substrate [122]. Curcumin analogs (i.e. 2,6-dibenzylidenecyclohexanone, 2,5-dibenzylidenecyclopentanone, and 1,4-pentadiene-3-one) are also shown to exert inhibitory effects on GSTs. However, their inhibitory effects on GTS A1-1, GTS M1-1, and GST P1-1 are smaller compared to curcumin [123]. Galangin is a flavonoid that suppresses the cellular activity of GST P1-1 at a concentration of 25 μM in GST P1-1 transfected MCF-7 breast cancer cells. Quercetin, kaempferol, and eriodictyol are other flavonoids that moderately inhibit the activity of GST P1-1. Most flavonoids (mainly quercetin and luteolin) are shown to inhibit GS-X pump transport. However, flavonoids without a C2–C3 double bond (e.g. catechin and eriodictyol) do not suppress the activity of the GS-X pump [124].

### Epigenetics

Epigenetic modification indicates some reversible changes in the expression of genes without causing changes in the sequence of DNA [125]. Mechanisms involved in epigenetics are capable of driving acquired cancer resistance against chemotherapeutic drugs. Epigenetic alterations occur at a high rate in tumors, leading to the diverse patterns of gene expression that cause drug resistance [126]. Since drug resistance can be reversed and it shows rapid kinetics and absence of genetic mutations, epigenetic mechanisms may be involved in insensitivity to drugs [127]. Epigenetic processes form different states of transcription which lead to a dynamic heterogeneous nature in the population of tumor cells [127]. As already mentioned, there are a number of mechanisms leading to epigenetic alterations, among which the role of miRNAs in the development of drug resistance in cancer is greatly investigated [128–130]. Therefore, targeting epigenetic changes and miRNAs with polyphenols may be a potential approach to overcome cancer drug resistance.

#### Resveratrol

DNA methylation by resveratrol has been explored by a very limited number of studies. Zadi Heydarabad and colleagues showed that DNA methylation of BAX and BCL2 genes in a T-cell acute lymphoblastic leukemia cell line, CCRF-CEM, can be detected after resveratrol treatment by a methylation-specific polymerase chain reaction technique. They suggested that this observation might explain the effect of resveratrol in sensitizing ALL cells to apoptosis [131]. In another study, the effect of resveratrol on retinoic acid resistance in the anaplastic thyroid cancer cell line THJ-11T and the human medulloblastoma UW228-2 cell line showed that resveratrol not only demethylates the cellular retinoic acid binding protein 2 (CRABP2) promotor but also decreases the amounts of some DNA methyltransferases such as DNMT1, 3A, and 3B [132]. Despite these studies, Zadi Heydarabad et al. [133] in another investigation found no relation between resveratrol and human multidrug resistance gene 1 (MDR1) methylation in the CCRF-CEM cell line; therefore, other drug resistance-related genes should be examined to clarify whether resveratrol is involved in DNA methylation-related drug resistance.

From another point of view, resveratrol induces apoptosis and enhances chemosensitivity to Adriamycin in MCF-7 breast cancer cells. It has been shown that the mentioned effects of resveratrol are inhibited by modulation of miR-122-5p, which is a critical suppressor. Moreover, modulation of miRNA by inhibitors or mimics of miR-122-5p demonstrate that miR-122-5p is involved in the regulation of CDKs (i.e. CDK2, CDK4, and CDK6) and anti-apoptotic factors (e.g. BCL-2) following resveratrol treatment [77].

#### Quercetin

Quercetin with a concentration of 5 μM improves the sensitivity of osteosarcoma 143B cells to cisplatin. Furthermore, it upregulates miR-217 expression while downregulating the expression of KRAS, which is the miR-217 target. Moreover, knockdown of miR-217 is shown to hinder quercetin-induced increased sensitivity to cisplatin. Therefore, quercetin’s ability to enhance cisplatin sensitivity is modulated by the miR-217-KRAS axis [134]. In the U-87 MG glioblastoma cell line, curcumin increases expression of miR-146a and enhances temozolomide-induced apoptosis. However, miR-146a inhibits the enhanced anti-tumor action of temozolomide which is induced by curcumin. Meanwhile, upregulation of miR-146a inhibits the activation of NF-κB and increases apoptosis in cells treated with temozolomide [135]. It seems that quercetin’s ability is not limited to microRNA regulation and DNA methylation and histone modifications can also be affected by this beneficial agent [136, 137]; however, these effects have not been examined in cancerous cells, and thus further investigations might expand the range of quercetin’s potential application.

#### Curcumin

According to evidence, curcumin is one of the most important polyphenols in regulating epigenetic alterations of cancerous cells [73, 74]; nonetheless, its efficacy in sensitizing tumoral cells to chemotherapeutic drugs is still controversial. In an in vitro study on the SiHa cell line, Roy and Mukherjee found that curcumin increases the effect of cisplatin on cervical cancer cells through several mechanisms including inhibition of histone deacetylase 1 (HDAC1) [138]. Royt et al. also confirmed that histone deacetylase can be decreased after curcumin treatment in the MCF-7 (ER positive) cell line [139]. Despite the small number of studies on DNA methylation and histone modification after curcumin treatment, the role of this agent in microRNA regulation has been intensively studied. Resistance to cisplatin in the A2780cp ovarian cancer cell line can be reduced indirectly by curcumin: demethylation in the promoter region of MEG3 occurs after curcumin usage, which leads to the down-regulation of miR-214. This microRNA is able to establish chemoresistance through increasing the capability of extracellular vesicles [140]. Decreased resistance to is also observed after treating Adr-resistant MCF-7 cells with curcumin-encapsulated liposomes [141]. Curcumin decreases the resistance to Adriamycin via altering the expression of some microRNAs including miR-29b-1-5p [141]. Overcoming Adriamycin-resistance has also been examined in human acute myeloid leukemia cells (HL-60) [142]. It seems that miR-20a-5p mediates the anti-resistant effects of curcumin in these cells and, overall, the HOTAIR/miR-20a-5p/WT1 pathway is the reason that curcumin can sensitize AML cells to Adriamycin (in vitro and in vivo) [142].

microRNA-27a is another microRNA whose down-regulation can result in lower resistance to 5-fluorouracil in SW-480 colon cancer cells [143]. Curcuminoids (2.5–10 μg/mL) disrupt the axis of miR-27a-ZBTB10-Sp and thereby suppress the expression of multidrug resistance protein (MDR1) [143]. Paclitaxel resistance, which is considered as a major issue in the treatment of aggressive non-small-cell lung cancers, is detected to be suppressed through the axis of mirRNA-30c/MTA1. The metastasis-associated gene 1 (MTA1) gene is reduced in these cells due to the up-regulation of microRNA-30c by curcumin (in vitro) [144].

### Cell death

Similar to cell growth and division, a key component of homeostasis is programmed cell death. Apoptosis, necrosis, and autophagy are three types of programmed cell death that are involved in development [145]. Several studies have been conducted on the underlying mechanisms of apoptosis in the past two decades. These investigations demonstrate that apoptosis involves different signaling pathways which are associated with survival pathways and change the phenotype of cells, such as drug resistance [146]. Apoptosis is a programmed cell death that removes aged and damaged cells from the body. In cancer, apoptotic signaling is dysregulated. Indeed, anti-apoptotic pathways are activated in cancer cells, which results in uncontrolled proliferation of cells, leading to drug resistance and tumor recurrence [147]. Autophagy is a mechanism of homeostatic cellular recycling which is involved in the response to therapeutic and metabolic stresses. By autophagy, the body tries to maintain or restore the homeostasis of metabolism via catabolic lysis [148]. Evidence has shown that cancer therapeutic approaches are capable of inducing autophagy. Meanwhile, autophagy is demonstrated to enhance the survival of tumor cells and lead to therapy resistance in some cases [149].

Gefitinib is an epidermal growth factor receptor (EGFR) tyrosine kinase inhibitor (TKI) which is used for treating patients with NSCLC. However, cases with wild-type mutations of KRAS and EGFR are resistant to this therapeutic agent. Curcumin is shown to increase the anti-tumor effects of gefitinib against the H1299 and H157 gefitinib-resistant NSCLC cell lines. Treating these cells with the combination of curcumin and gefitinib leads to the induction of autophagy and autophagy-mediated apoptosis. Pharmacological inhibitors of autophagy, 3-MA, or Bad A1 are also shown to reverse the synergistic effect of curcumin and gefitinib [150]. Resveratrol exerts anti-tumor effects against human oral cancer CAR cells while showing low toxicity in normal oral cells. As evidenced by acridine orange (AO) and monodansylcadaverine (MDC) staining, resveratrol treatment leads to the formation of autophagic vacuoles and acidic vesicular organelles. Furthermore, resveratrol induces expression of autophagy-related genes at the mRNA level, including Beclin-1, Atg5, Atg12, and LC3-II. It also leads to apoptosis as evidenced by DNA condensation or DNA fragmentation. Resveratrol-induced cleavage of caspase-3 and -9 as well as apoptosis is reduced by Z-VAD-FMK, a pan-caspase inhibitor. Moreover, inhibitors of PI3K class III and AMPK (3-MA and compound c, respectively) lead to inhibition of autophagic vesicle formation and protein levels of LC3-II [151].

In a methotrexate (MTX)-resistant osteosarcoma cell line (U2-OS/MTX300), quercetin plays antitumor roles without showing cross-resistance with MTX. As evidenced by fluorescence staining and cytometry, quercetin induces apoptosis in these cells. Apoptosis is paralleled by mitochondrial cytochrome c release to the cytosol, reduced mitochondrial membrane potential, Akt dephosphorylation, upregulation of caspase-3 and Bax as well as downregulation of p-Bad and Bcl-2. Notably, constitutive activation of Akt hinders quercetin-mediated Akt and Bad dephosphorylation as well as degradation of poly(ADP-ribose)polymerase (PARP) [152]. In human leukemic multidrug-resistant K562/Adriamycin (ADR) cells, the combination of Adriamycin and quercetin leads to enhanced cytotoxicity. Quercetin treatment enhances the apoptosis of tumor cells. Indeed, it increases the expression of Bcl-2-associated death promotor, Bcl-2-interacting mediator of cell death, and Bcl-2-associated X protein. Also, it reduces the potential of mitochondrial membrane potential as well as expression of Bcl-2 while activating caspase-3, -8, and -9 [153]. In cisplatin-resistant oral cancer CAR cells, EGCG induces apoptosis and autophagy. EGCG increases the protein levels of cleaved caspase-3 and -9 as well as their activities. Additionally, it enhances the protein levels of Bax, Beclin-1, LC3B-II, Atg5, Atg7, and Atg12. Meanwhile, it decreases the Bcl-2 expression, STAT3 phosphorylation of Tyr705, and Ser473 (phosphorylated AKT). The expression of multidrug resistance 1 (MDR1) is also suppressed by EGCG at gene and protein levels [154] (Table 1).

**Table 1:** Polyphenols that exert an effect on autophagy to increase the sensitivity of tumor cells to chemotherapeutic drugs

Compound	Chemotherapeutic drug	Result	Refs.
Curcumin	Gefitinib	Enhances the efficacy of the drug and overcomes the EGFR-TKI resistance in NSCLC patients with wild-type EGFR and/or KRAS mutation	[150]
	5-Fluorouracil	Exerts synergistic effect with the chemotherapeutic drug by impairing AMPK/ULK1-dependent autophagy	[155]
	Docetaxel	Leads to induction of apoptosis and autophagy through PI3K/AKT/mTOR pathway	[156]
Resveratrol	Cisplatin	Induces autophagic and apoptotic death in drug-resistant oral cancer cells	[151]
	Gefitinib	Overcomes drug resistance while inducing apoptosis, autophagy, and senescence in PC9/G NSCLC cells	[157]
EGCG	Cisplatin	Increases sensitivity of CAR cells, apoptosis, and autophagy by AKT/STAT3 pathway	[154]
Apigenin	Cisplatin	Inhibits growth of drug-resistant colon cancer cells while inducing autophagy	[158]
Liquiritin	Cisplatin	Induces apoptosis and autophagy in drug-resistant gastric cancer cells	[159]
GL-V9	Adriamycin	Reverses drug resistance by blocking JNK2-related protective autophagy in HCC	[160]

### Alterations in DNA repair

DNA damage response (DDR) is a collection of mechanisms involved in detecting DNA damage and signaling it, which lead to either DNA repair processes or cell death pathways [161, 162]. DDR plays a protective role for the human genome against damage through removing errors and inhibiting mutation insurgence under physiological conditions. However, DNA repair systems work in favor of tumor cells following treatment with DNA damaging agents, causing failure in treatment [163].

Excision repair cross-complementation group 1/xeroderma pigmentosum group F (ERCC1/XPF) is an endonuclease that is involved in DNA damage repair following cisplatin treatment. The green tea polyphenol (–)-epigallocatechin-3-gallate (EGCG) is shown to inhibit the activity of ERCC1/XPF and DNA repair, leading to enhanced sensitivity of non-small cell lung cancer cell lines to cisplatin. Moreover, in vivo examination of this agent in 20 female athymic mice showed that EGCG octaacetate, an EGCG prodrug, improves the efficacy of platinum-based chemotherapy [164]. Thymidine phosphorylase is an enzyme in the pyrimidine salvage pathway which hinders DNA damage-induced cell death in cancer cells [165]. Curcumin treatment reduced the expression of thymidine phosphorylase at mRNA and protein levels in non-small-cell lung cancer. Furthermore, it downregulated ERCC1 by inactivating MKK1/2-extracellular signal-regulated kinase (ERK1/2). A similar study also indicated that demethoxycurcumin is able to decrease the resistance to cisplatin by means of downregulating ERCC1 in non-small cell lung cancer (both in vitro and in vivo) [166].

Evidence indicated that curcumin increases the cisplatin sensitivity of lung cancer cells by inactivating ERK1/2 and reducing protein levels of ERCC1 and thymidine phosphorylase [165]. Cisplatin exerts its antitumor effects through the formation of intra- and inter-strand cross-links of DNA, leading to blockade of DNA replication. Fanconi anemia (FA)/BRCA is a repair pathway for DNA cross-link damage and modulates the resistance of cells to DNA cross-link agents, such as cisplatin. In cisplatin-resistant lung adenocarcinoma (A549/DDP) cells, curcumin increases the antitumor effects of cisplatin. Furthermore, curcumin decreases the cisplatin-induced mono-ubiquitination of FANCD1 and formation of nuclear foci. This implies that curcumin-induced enhanced cisplatin sensitivity is mediated by suppression of the FA/BRCA pathway [167]. BRCA1 and RAD51 are two proteins leading to homologous recombination (HR) and thereby repair of double-strand DNA breaks [168].

Resveratrol enhances the antitumor effects of cisplatin in MCF-7 and chemo-resistant MCF-7 (MCF-7R) breast cancer cells. Data demonstrated that resveratrol at a concentration of 100 μM reduces the protein levels of Rad51 and transcript levels of components of the HR initiation complex. It has been observed that following 48 h of DNA damage induced by cisplatin, Rad51 protein levels are increased. However, resveratrol suppresses the upregulation of Rad51. Also, resveratrol sustains the phosphorylation of histone H2AX at serine 139, implying the inhibitory effect of resveratrol on the repair of double-strand breaks (DSBs) [169]. Furthermore, examinations on patient-derived glioblastoma-initiating cell lines have clarified another aspect of resveratrol’s DDR-associated functions: enhancing the cytotoxicity of temozolomide through DNA double-stranded breaks/pATM/pATR/p53 pathway activation [170].

### Cancer stem cells

Cancer stem cells (CSCs) are a class of tumorigenic cells that are mostly known for their self-renewal and multipotency features and make up less than 1% of the cells existing within a tumor [171, 172]. Investigations on almost all types of cancer have revealed that CSCs are responsible for decreasing the tumor response to both chemo- and radiotherapy [173–184]. It seems that slowing down the cell cycle, having anti-apoptotic machinery, a high capacity for repairing DNA damage, the potency of establishing a proper environment for cancer growth, and stemness features are the main reasons why CSCs provide resistance to our common therapies [171, 172, 185, 186]. Notch, Wnt, STAT3, PI3K/Akt, and NF-kB signaling pathways along with protective autophagy, metabolic plasticity, and oxidative modulators are some other helpers of CSCs in this process [186, 187]. Overall, targeting CSCs is a suitable approach for decreasing tumor resistance and increasing the efficacy of our common therapies.

Considering polyphenols for targeting this population of cells has recently given a new insight for treating resistant cancers. A line of research conducted on curcumin showed the anti-CSC characteristics of this agent in colon [188], pancreatic [189], liver [190], breast [50], and brain [191] cancers. Curcumin treatment administered for cancerous cells of the colon resulted in lower levels of CSC markers such as CD44, CD133, and CD24 and lower ability of CSCs to form a sphere [188]. Additionally, curcumin triggers apoptosis in CSCs either administered alone or with irinotecan (CPT-11) and thereby decreases resistance to this chemotherapeutic drug [188]. Decreasing the stemness features of CSCs through inhibiting the enhancer of zeste homolog-2 (EZH2) subunit of polycomb repressive complex 2 (PRC2) is also attributed to curcumin [189, 192]. Inhibiting a long non-coding RNA named PVT1 is how curcumin sensitizes pancreatic cancer cells to gemcitabine [189]. Other than that, stemness-related genes including Nanog, Sox2, and Oct4 are also prone to be affected by curcumin [50]. Another mechanism by which curcumin decreases chemoresistance is its ability to decrease antiapoptotic protein levels and increase proapoptotic protein levels in CSCs [193]. The former proteins include Bcl-2 and Bcl-w and the latter ones include Bax, Bak, Bad, Bik, and Bim. In this way, the resistance of breast cancer to mitomycin C can be diminished [193]. Nanomedicine can also be effective in treating brain cancer with curcumin [191]. Curcumin-loaded nanoparticles grafted with anti-aldehyde dehydrogenase not only increased the permeability of curcumin through the brain-blood barrier but also provided a steady release of this polyphenol [191]. An in vivo study conducted by Zhou et al. [194] also demonstrated that curcumin increases the sensitivity of breast cancer cells to mitomycin C. In this study, ATP-binding cassette (ABC) transporters ABCG2 and ABCC1 acted as mediators of curcumin’s effects on breast cancer stem cells, which were reduced after the combinatorial treatment [194].

Epigallocatechin-3-gallate is another member of the polyphenol family which is also able to reverse some of CSC characteristics including renewal and migration in nasopharyngeal cancer cells [195]. Baicalin is a flavone whose role in EMT has been investigated on an osteosarcoma cell line: it can inhibit the EMT-inducing transcription factors Snail1 and Slug and thereby reduce the anoikis-related resistance [196]. Additionally, baicalin inhibits the PI3K/Akt/NF-κB pathway, which leads to EMT reversion and a decrease in cisplatin resistance of lung adenocarcinoma cells (in vivo and in vitro) [197].

An in vivo study conducted by Toden and colleagues also corroborated the anti-resistance effects of EGCG on cancer stem cells. They observed that 5FU sensitivity could be achieved when EGCG suppressed colorectal cancer stem cells through affecting the Notch signaling pathway and increasing the levels of tumor suppressive microRNAs [198].

### Reactive oxygen species

Through altering the hemostasis of redox, cancer cells enhance chemoresistance. Different processes are involved in chemoresistance mediated by redox which includes increased progression of the cell cycle, autophagy mediated by endoplasmic reticulum stress, increased numbers of cancer stem-like cells, and enhanced conversion to metastasis [199]. ROS exert several functions in cancer cells, such as regulating proliferation, apoptosis, and survival. Compared to normal and non-multidrug-resistant cancer cells, ROS levels and the activity of antioxidant enzymes are increased in cancer cells that are resistant to chemotherapeutic agents. Therefore, changes in ROS levels may have a greater impact on multidrug-resistant cancer cells. Studies have shown that agents modulating the generation of ROS are potentially useful for treating patients with multidrug-resistant cancer [200].

The thioredoxin (Trx) system has three key members which are NADPH, Trx, and thioredoxin reductase (TrxR). This system, which is involved in the regulation of redox, has been shown to play a role in the development and progression of cancer. Indeed, high expression levels of Trx and TrxR in cancer cells lead to drug resistance [201]. Ai and colleagues have designed 21 ligustrazine-curcumin hybrids (10a-u) and reported that compound 10d suppresses the proliferation in both drug-resistant and drug-sensitive lung cancer cells. It was observed that 10d inhibited the Trx/TrxR system while enhancing the intracellular accumulation of ROS as well as apoptosis [202]. In another study by Zhou et al. an analog of curcumin, (1E,4Z,6E)-5-hydroxy-1-(4-hydroxy-3-methoxyphenyl)-7-(5-methylfuran-2-yl)hepta-1,4,6-trien-3-one (2a), suppressed the growth of cisplatin-resistant lung cancer A59 cells. Moreover, A549/CDDP cells pretreated with 2a show sensitivity to cisplatin while TrxR activity is suppressed in them. 2a consequently increases the intracellular accumulation of ROS and decreases glutathione (GSH) and the GSH/GSSG ratio, indicating the shift of intracellular redox balance to an oxidative state [203].

Nuclear factor erythroid 2-related factor 2 (NRF2) is a transcription factor that is traditionally found to regulate the protective mechanisms against oxidative stress [204]. Recent investigations have shown that this transcription factor is also involved in the development and progression of cancers as well as chemoresistance [205–210]. Therefore, NRF2 is reported to be a potential candidate in overcoming cancer chemoresistance [211]. Zhang et al. [212] reported that curcumin induces the deficiency of Nrf2, which, in turn, changes the ratio of Bcl-2 associated X protein/Bcl-2 expression. Consequently, curcumin treatment results in apoptosis induction in HCT-8/5-Fu cells and reverses the multidrug resistance in colorectal cancer cells. Curcumin also chemosensitizes head and neck squamous carcinoma cells to cisplatin in vitro through targeting Nrf-2 and pSTAT3 signaling pathways [213]. In pancreatic cancer cells, resveratrol inhibits the nutrient-deprivation autophagy factor-1 (NAF-1) via activating the Nrf2 pathway and accumulating intracellular ROS [214]. Expression levels of Nrf2 as well as NADPH and heme oxygenase-1, target proteins of Nrf2, are higher in tamoxifen-resistant MCF-7 (MCF-7/TAM) cells compared to MCF-7 cells. EGCG is reported to significantly increases the sensitivity of MCF-7/TAM cells to tamoxifen while decreasing the mRNA and protein levels of Nrf2. Using siRNA of Nrf2 reverses the tamoxifen resistance in MCF-7/TAM cells. Furthermore, combined treatment with EGCG and Nrf2 siRNA results in a synergic Nrf2 downregulation and reversal of tamoxifen resistance [215].

The expression of androgen receptor (AR) is significantly associated with poor prognosis of glioblastoma patients [216]. Moreover, AR leads to the development of resistance against temozolomide. The curcumin analog ALZ003 is able to induce ubiquitination of AR by FBXL2, resulting in its degradation. ALZ003 suppresses the survival of both temozolomide-sensitive and -resistant glioblastoma cells in vitro and in vivo. Treating glioblastoma cells with ALZ003 also leads to lipid peroxidation, ROS accumulation, and inhibition of glutathione peroxidase (GPX) 4, which are indicators of ferroptosis. Regarding the in vivo effect of this treatment, Chen and colleagues transplanted temozolomide-sensitive or -resistant glioblastoma U87MG cells into mouse brain. They observed that ALZ003 suppressed the proliferation of tumor cells as well as increasing their survival [216] (Table 2).

**Table 2:** Modulating ROS to overcome cancer drug resistance

Mode of action	Compound	Application	Refs.
TrxR	2a, curcumin analog^a^	Sensitizes A549 cells to cisplatin	[203]
	10d^b^	Effective on drug-sensitive (A549, SPC-A-1, LTEP-G-2) and drug-resistant (A549/DDP) lung cancer cells	[202]
Nrf2	Curcumin	Mediates cisplatin chemoresistance in head and neck squamous cell carcinoma	[213]
		Reverses multidrug resistance in the HCT-8/5-Fu human colorectal cancer	[212]
	Resveratrol	Enhances the sensitivity of pancreatic cancer cells to gemcitabine	[214]
	Chrysin	Reduces doxorubicin resistance by down-regulating Nrf2 signaling pathway in BEL-7402/ADM cells	[217]
GPX	ALZ003, curcumin analog	Suppresses growth of temozolomide-resistant glioblastoma	[216]

^a^(1E,4Z,6E)-5-hydroxy-1-(4-hydroxy-3-methoxyphenyl)-7-(5-methylfuran-2-yl)hepta-1,4,6-trien-3-one (2a)

^b^Active compound of 21 ligustrazine-curcumin hybrids (10a-u)

### Epithelial to mesenchymal transition

Epithelial to mesenchymal transition or EMT is a dedifferentiation process whose target is to prepare epithelial cells for migration and metastasis [186]. Diverse molecular alterations cause the transformation of polar epithelial cells into multipolar and motile mesenchymal cells [218]; therefore, epithelial genes containing E-cadherin, ZO-1, and occludin should be decreased in cells undergoing EMT while mesenchymal genes containing N-cadherin, vimentin, and fibronectin ought to be increased [218]. Similar to every other cellular process, signaling pathways have a pivotal role in EMT; these pathways encompass transforming growth factor beta (TGFβ), Wnt, Notch and Hedgehog [219]. The mechanism by which EMT contributes to the resistance of several cancer types is mainly related to CSCs [220]. This association was suggested due to the similarity of signaling pathways involved in EMT and CSCs. To our knowledge, CSCs probably recall EMT by affecting the non-CSC cells of the tumor [186]. The ATP-binding cassette (ABC) transporters seem to be greatly involved in CSC-induced EMT of tumor cells. Investigations on resistant tumor cells undergoing EMT reveal the higher expression of these transporters [221]. As well as CSCs, EMT is also susceptible to the ingredients of the tumor microenvironment including cancer-associated fibroblasts (CAF) and hypoxia [220, 222].

For targeting EMT in cancer cells, curcumin is confirmed to be a suitable option. It decreases 5-fluorouracil-resistance by means of affecting the TET1-NKD2-WNT axis and thereby hindering EMT [223]. MicroRNAs are other mediators of curcumin’s effects; miR-200b, miR-200c, miR-141, miR-429, and miR-101 are anti-EMT miRNAs that are upregulated after curcumin treatment [192]. In colorectal cancer cells, this effect aids the reduction of 5-fluorouracil resistance [192]. Furthermore, in colorectal cancer calls, curcumin increases the markers of epithelial cells including E-cadherin when utilized with irinotecan [224]. Regulating the TGF-β/Smad2/3 signaling pathway for decreasing the resistance to oxaliplatin through EMT inhibition is another mechanism of curcumin’s actions in colorectal cancer cells [225]. H19 is a long noncoding RNA with the role of establishing tamoxifen resistance in breast cancer cells, which can be impacted by curcumin [226]. H19 overexpression is responsible for EMT in breast cancer cells, which can be reversed after curcumin treatment [226].

In addition to curcumin, resveratrol might also be a suitable option for overcoming resistance through EMT inhibition [227]. A variety of ways have been revealed for resveratrol to affect EMT: “reactivating p53 and inducing miR-145 and miR-200c” [228], modulating the connection between SIRT1 and β-catenin [229], and modulating the PTEN/Akt signaling pathway [230]. EMT inhibition is also possible by quercetin. Quercetin decreases the resistance to docetaxel in prostate cancer cells by affecting some mechanisms including EMT [231]. NF-κB p65 inactivation is another mechanism that is used by epigallocatechin-3-gallate to inhibit EMT in nasopharyngeal cancer stem cells [195]. EGCG, which is a green tea polyphenol, seems to be effective against CSCs of nasopharyngeal carcinoma and inhibits some of their features including EMT [232]. Lin et al. [232] demonstrated this effect by measuring EMT markers such as Snail, vimentin, and E-cadherin after EGCG treatment. Geraniin is another member of the polyphenol family which can be found in *Phyllanthus amarus *[233]. Elevating the levels of E-cadherin and decreasing the levels of Snail are two effects of this agent on lung cancer cells. After using geraniin, anoikis resistance is decreased in these cells through inhibition of EMT [233].

## Conclusions

**Fig. 3:**
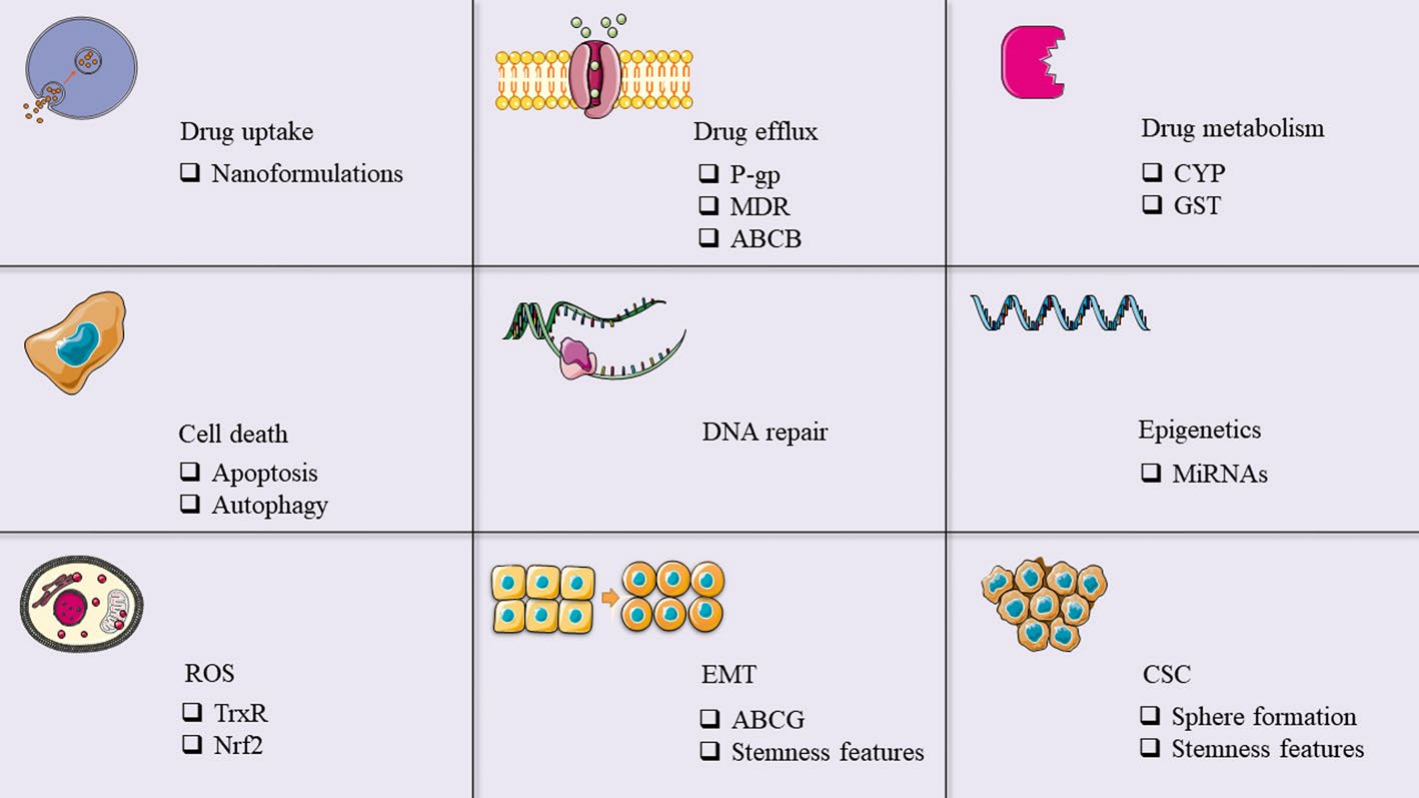
Mechanisms involved in cancer drug resistance in which polyphenols may play a role

Our knowledge of the anti-cancer effects of polyphenols has remarkably expanded since the discovery of these beneficial agents, which is why the understanding of the underlying mechanisms by which they work is essential. Accumulative investigations have clarified how these natural compounds are able to inhibit cancer hallmarks such as proliferation, apoptosis, inflammation, etc. But what if polyphenols have a more significant ability that would assist us through overcoming cancer? Resistance to the common therapies involving chemo- and radiotherapy is our most important difficulty in restricting the progression of cancer cells and decreasing the number of cancer-related deaths. In this regard, we have taken a look at the polyphenols’ mechanisms of action which are specific to overcoming the resistance of different cancers (summarized in Fig. 3). According to the discussed studies, affecting the gene expression and signaling pathways associated with cancer stem cells is an important aspect of polyphenols’ effects which makes them capable of inhibiting EMT, oxidative stress, and other stemness features to reduce the resistance. Increasing drug uptake by tumor cells, decreasing drug metabolism by enzymes (e.g. cytochromes and glutathione-S-transferases), and reducing drug efflux are some of the mechanisms by which polyphenols increase the sensitivity of cancer cells to chemotherapeutic agents. Polyphenols also affect other targets for overcoming chemoresistance in cancer cells, including cell death (i.e. autophagy and apoptosis), EMT, ROS, DNA repair processes, cancer stem cells, and epigenetics (e.g. miRNAs). So far, only a few clinical trials have been performed on the role of polyphenols in overcoming cancer drug resistance. One of them is a phase II study of resveratrol (SRT501) with bortezomib in patients with relapse of refractory multiple myeloma. This study has shown that this therapeutic approach has a low safety profile while showing minimal efficacy [234]. Another clinical trial is a phase I/II study of curcumin and gemcitabine in patients with gemcitabine-resistant pancreatic cancer. This study, performed on 21 patients, shows that combined therapy with gemcitabine and 8 g of oral curcumin daily is a safe and feasible treatment for patients and further investigations should be carried out to define its efficacy [235]. Taken together, the developments in our understanding of these agents have revealed the wide range of their effects against treatment resistance, but still, the lack of sufficient human research is holding us back from using polyphenols widely in clinical practice.

## Data Availability

Not applicable.

## Abbreviations

EGCG: Epigallocatechin gallate
miRNA: MicroRNA
DOX: Doxorubicin
AML: Acute myeloid leukemia
p-gp: P-glycoprotein
ABC: ATP-binding cassette
MRP1: Multidrug resistance protein 1
BCRP: Breast cancer resistant protein
5-FU: 5-Fluorouracil
ABCB4: ATP binding cassette subfamily B member 4
GGTs: Gamma-glutamyl transferases
TPMTs: Thiopurine methyltransferases
GSTs: Glutathione-S-transferases
DPDs: Dihydropyrimidine dehydrogenases
UGTs: Uridine diphospho-glucuronosyltransferases
GGTP: Gamma-glutamyl transpeptidase
TKI: Tyrosine kinase inhibitor
EGFR: Epidermal growth factor receptor
NSCLC: Non-small-cell lung cancer
MTX: Methotrexate
MDR1: Multidrug resistance 1
